# Supplementary material for: Machine learning model to predict the adherence of tuberculosis patients experiencing increased levels of liver enzymes in Indonesia
Source: PLoS One. 2025 Jan 24;20(1):e0315912. doi: 10.1371/journal.pone.0315912 (PMC11760577; doi:10.1371/journal.pone.0315912)
Supplement: Attachment — Submitted filename: Response to the reviewer.docx [file pone.0315912.s001.docx]

Dear Editor

Thank you for the review after we revised our manuscript (PONE-D-24-35376R1).

Below is our response to the reviewer suggestion.

We look forward to hearing from you regarding our submission. We would be glad to respond to any further questions and comments that you may have

Best regards,

Corresponding letter

Dyah Aryani Perwitasari

**Response to the editor and reviewer**

Journal Requirements:

Please review your reference list to ensure that it is complete and correct. If you have cited papers that have been retracted, please include the rationale for doing so in the manuscript text, or remove these references and replace them with relevant current references. Any changes to the reference list should be mentioned in the rebuttal letter that accompanies your revised manuscript. If you need to cite a retracted article, indicate the article’s retracted status in the References list and also include a citation and full reference for the retraction notice.

*Response*

1. The reference list is based on the cited reference in the body of text
2. We do not have any retracted publication

**Comments to the Author**

1. If the authors have adequately addressed your comments raised in a previous round of review and you feel that this manuscript is now acceptable for publication, you may indicate that here to bypass the “Comments to the Author” section, enter your conflict of interest statement in the “Confidential to Editor” section, and submit your "Accept" recommendation.

Reviewer #1: All comments have been addressed

2. Is the manuscript technically sound, and do the data support the conclusions?

The manuscript must describe a technically sound piece of scientific research with data that supports the conclusions. Experiments must have been conducted rigorously, with appropriate controls, replication, and sample sizes. The conclusions must be drawn appropriately based on the data presented.

Reviewer #1: Partly

3. Has the statistical analysis been performed appropriately and rigorously?

Reviewer #1: Yes

4. Have the authors made all data underlying the findings in their manuscript fully available?

The [PLOS Data policy](http://track.editorialmanager.com/CL0/http:%2F%2Fwww.plosone.org%2Fstatic%2Fpolicies.action%23sharing/1/010f01936916b7a3-9ad5d5b5-9e81-4221-b75f-ac9b176cdace-000000/F2cZ2QR1QHL6c4JYvpg5x8xGnFo4FqX3yKV-QYUvwsg=187) requires authors to make all data underlying the findings described in their manuscript fully available without restriction, with rare exception (please refer to the Data Availability Statement in the manuscript PDF file). The data should be provided as part of the manuscript or its supporting information, or deposited to a public repository. For example, in addition to summary statistics, the data points behind means, medians and variance measures should be available. If there are restrictions on publicly sharing data—e.g. participant privacy or use of data from a third party—those must be specified.

Reviewer #1: Yes

5. Is the manuscript presented in an intelligible fashion and written in standard English?

PLOS ONE does not copyedit accepted manuscripts, so the language in submitted articles must be clear, correct, and unambiguous. Any typographical or grammatical errors should be corrected at revision, so please note any specific errors here.

Reviewer #1: Yes

*Response* : Thank you for accepting my revisions

6. Review Comments to the Author

Please use the space provided to explain your answers to the questions above. You may also include additional comments for the author, including concerns about dual publication, research ethics, or publication ethics. (Please upload your review as an attachment if it exceeds 20,000 characters)

Reviewer #1: Dear authors, thanks for sending back your revised manuscritp. It has improved after your revision. However, I believe it would benefit from one last very careful review of grammar and wording, as there are some repeated small inconsistences - e.g., in abstract, you mention "adverse reaction", while in text, "adverse drug reaction". Moreover, the letter style in the figures differ from the text.
And just one question: I did not see any grading criteria for the adverse drug reaction - if you did not use any, please specify in methods.

*Response:*

Thank you for the comments and suggestion.

We already revised the adverse reaction into adverse drug reaction.

We already adjust the letter style in the figures.

We did not use the grading criteria, and put this statement in the method :

We did not use the grading criteria for the adverse drug reaction, because for the laboratory data we used the continuous data. For the patients’ symptoms, it was collected based on the patients’ experienced.

While revising your submission, please upload your figure files to the Preflight Analysis and Conversion Engine (PACE) digital diagnostic tool, [https://pacev2.apexcovantage.com/](https://track.editorialmanager.com/CL0/https:%2F%2Fpacev2.apexcovantage.com%2F/1/010f01936916b7a3-9ad5d5b5-9e81-4221-b75f-ac9b176cdace-000000/kg1pNdKk42ejr_I_9Znv3jrwlxOtJUtinsUps3o44OA=187). PACE helps ensure that figures meet PLOS requirements. To use PACE, you must first register as a user. Registration is free. Then, login and navigate to the UPLOAD tab, where you will find detailed instructions on how to use the tool. If you encounter any issues or have any questions when using PACE, please email PLOS at [figures@plos.org](mailto:figures@plos.org). Please note that Supporting Information files do not need this step.

*Response :*

We already proceed our figures in PACE and upload separated in the system
